# Scabbies

**Published:** 1865-01

**Authors:** James C. White


### SELECTED.

### SCABIES.

[Read before the Boston Society for Medical Improvement, and communicated to the *Boston Medical and Surgical Journal.*]

By JAMES C. WHITE, M. D.

In the days of our fathers scabies was a diseases of frequent occurrence and was the pest of the village school, but it gradually died out under more cleanly ways of living, until in our generation it has become so rare that many physicians do not recognize it even when called upon to treat it. The frequent mistakes in diagnosis which arise through this want of familiarity, the importance of the immediate recognition of its existence in a family, and its marked increase among us within a few years, lead me to ask the attention of the Society to some points connected with its nature, diagnosis and treatment as they were observed by me in the clinic of Prof. Hebra.

Three forms of the itch insect are met with upon the skin, viz., the female, male and young. The mature female is discernible with the naked eye as a speck, 1-4 line in length and

1-6 line in breadth. It is of a white color, and resembles in form a tortoise shell, with an arched back and flat belly. The male is only half the size of the female, and, until recently discovered and described by Boeck and Danielsson in cases of Norway itch, was unknown. In structure it differs but slightly from the female. * * * * It is not white and shining like the female, but black and compressed. The young possess but three pairs of legs, and in them no distinction of sex is noticeable. In order to become mature they undergo, according to some writers, three metamorphoses; according to others, only one. Whether all these changes are essential or not, it is at least a fact that the six-legged form may be found containing within itself a visible eight-legged one. After maturity the females cease to creep over the surface of the skin, and remain in new burrows till sought after by the males. One coitus must suffice to impregnate the entire ovary of the female, for after copulation she burrows deeper and deeper, and forms the long burrows to be more particularly described hereafter. The male never enters one of these true burrows where the eggs are found, but digs himself a shallow cell or seeks new fields for his sexual rambles. The female goes on her downward course obliquely through the epidermis, and leaves behind her each day a new laid egg and masses of fæcal matter. The direction taken must necessarily be oblique, for otherwise the rapid increase of the epidermis from below upwards and discharge of effete superficial layers would allow the escape of the eggs before the proper time. After 14 are thus deposited, the larva of the oldest, which has been gradually pushed up from below, becomes mature, and emerges from its birth-place through the opening made by the mother to undergo the processes above described, leaving behind its broken egg-shell. All stages of development may be witnessed in these eggs, from the amorphous form in which they are deposited, through segmentation, to the perfect young before it breaks its prison. The old female never leaves her hole, and sometimes wanders along for four inches beneath the surface. Fifty eggs and broken shells are sometimes counted in such burrows, although they never contain more than 14 larvæ at one time. If, however, a pustule should be formed the eggs are destroyed, but the female emerges at the surface near the edge of the pustule, and commences her descent anew. The whole time required for the young to reach maturity after impregnation is estimated at six weeks. How long the female may live is not known, but she probably dies at the extremity of her burrow after depositing all her eggs.

The symptoms produced by the presence of these minute animals are of two kinds—the direct or primary, those caused by the animal itself, and the indirect or secondary, those produced in consequence of the scratching. They are both subjective and objective.

*The Primary.*—We have already seen that the appearances on the skin must differ according to the age and sex of the parasite. The young and males make only a little pit or short burrow for purposes of metamorphosis or nutrition, and at most give rise to the formation of a minute vesicle. It is the impregnated female only which produces the characteristic pathognomonic sign of scabies, the burrow for the deposition of her eggs. These burrows, when closely observed, are found to be linear elevations, running an irregular course, with an uneven surface corresponding to the situation of the eggs within, and terminating at one extremity in a marked prominence, which is the old one at work. Bourgignon considered these irregularities of surface to be caused by openings in the course of the burrow through which the female emerged at night; but, as already stated, it is known that she never leaves her hole, and microscopic examination shows the eggs lying with the long axis perpendicular to that of the tunnel, and that no such communication with the surface above exists. It reveals, also, the presence of numerous reddish brown globular masses scattered throughout its whole length, which are the fæces of its inhabitant. In persons of cleanly habits the burrows are generally distinguishable by their white color, but in classes among which scabies most commonly occurs, they are mostly stained of a darker tint than the surrounding epidermis, from the absorption of dirt and coloring matters. In length they vary, averaging generally from one or two lines to half an inch, but in exceptional cases they may stretch out to three or four inches, although the eggs are much farther apart in such instances, as the number in each individual is probably limited to 40 or 50. The direction they take is slightly zigzag or curved, seldom perfectly straight. When fresh, a small vesicle often marks the point of entrance of the animal, and in other instances the whole burrow is lifted prominently above the surface by the exudation excited beneath it, which may be converted into a pustule and thus destroy the embryos. In rare cases a large papule may be formed beneath the burrow, over the summit of which it may be seen to run. The entrance of the tunnel generally remains open for the exit of the young and for the admission of air. When the disease has existed for a long time they may run into each other, or secondary appearances may show themselves, which obscure the typical forms and present to an inexperienced eye no sign of scabies.

As to the *subjective* symptoms produced by these minute creatures, the plain Saxon name of the disease indicates strongly enough their nature. It is *the Itch.* The sensation experienced by the patient is produced in a degree only by the old female. It is the depredations of the young and males rambling at will over the surface of the body and boring at random into the deeper and senstive layers of the epidermis, or into the upper surface of the papillary structure, which produce an itching so intolerable as to make a resort to scratching an imperative necessity. The scratching, when once excited, however, is not confined to the local irritation of the affected spot alone, but, owing to the intimate connection existing between the sensitive cutaneous nerves and the pleasurable sensations thus produced, is converted into a general application of the nails to large surfaces of the surrounding skin.

This itching, which is a primary and subjective symptom, leads us from cause to effect, to the *secondary* phenomena of the disease—the eruptive appearances of scabies. It is only necessary to assume that scratching is capable of producing upon a person harboring a colony of sarcoptes the same appearances as upon the skin of anybody else, and we may account for all the objective symptoms of this affection, with the exception of the burrows already described, without being obliged to consider the possibility of any such absurdities as a psora dyscrasy or the absorption of the irritating fæcal matter of the insect. Let any one scratch himself roughly on the breast or thigh and study the result. We first see broad red lines corresponding to the irritated parts; this is erythema. The follicles then begin to swell up in consequence of the exudation, or, in other words, a papular eruption appears. Carry on the scratching farther, and we cause an excoriation and hemorrhage, by which the tip of each papule is colored black by the dried blood, as in prurigo. Again, if we scratch or interfere with the process of an exudation, what is the result? a pustule appears. It is incorrect, then, to say that such and such forms of eruption are caused by this animal. It has nothing at all to do with them, except to cause an itching, from which they all result. The eczema and vesicles, papules and pustules are wholly caused by our nails and our clothes, and not by the parasite. In individuals who can not scratch, no such forms are met with. In the beginning of the disease the itching is very slight, but after a fortnight has passed, and the young are beginning to emerge and colonize fresh portions of the skin, it increases in intensity, and leads to more frequent indulgences in scratching, in consequence of which red spots or papules appear about the openings of the hair follicles. By the third week the itching has become so intense as to be no longer satisfied by superficial friction, and excoriation and a pruriginous eruption, tipped with bloody caps, bear witness to the execution effected by the nails. Finally vesicles, pustules, patches of eczema rubrum, and ecthyma, and depositions of pigment follow, and in some cases the extremities may be covered with crusts of the size of a copper cent, and with pigment spots, the seats of old scabs, which might be mistaken for syphilis. The eczema and pustules are at times sufficient to cause the neighboring lymph-glands to swell. These appearances are confined to certain regions of the body, as the chest, abdomen, thighs, and hands, not the parts where the burrows are in the greatest abundance, nor are they in proportion to their number, but those which are most easily accessible to the nails. Upon the neck, back, shoulders, and lower extremities very little eruption is found. Wherever constant pressure is made, there the appearances will be exaggerated. Those who sit all day, as shoemakers, tailors, and others, have the pustules and papules most developed on the buttocks, just as occurs in the axillæ of those who walk with crutches, and about the waist and under the garters of women. The appearances which the surface of the body presents in those who have had the itch a long time are most variable, and even long after the cause has disappeared, we may find an eruption which would serve as a model to illustrate almost every form of cutaneous disease.

A peculiar variety of scabies is met with in Norway, which is distinguished by the formation of huge crusts upon the surface of the skin like rupia, and by the presence of the parasite upon the face and even beneath the nails. Its frequent occurrence in that country and its rarity in other places, only some half dozen cases having been observed elsewhere, has led to the supposition that the disease was caused by some peculiar form of acarus, but Hebra has shown that the crust consists of dried epithelium, dead sarcoptes and their fæces, and that beneath, the whole epidermis is undermined by the swarms of animals, adults of both sexes, and young, living without burrows, which are identical with the ordinary variety. It was in such social colonies that the male was first discovered and described. This form leads often to serious complications, as immobility and great swelling of the limbs, as in elephantiasis, with severe pain and inflammation, and is at times confined to a circumscribed part of the body, while the rest of the surface is affected in the usual way. We seek in vain for a satisfactory explanation of this form of the disease. Hebra thinks that great uncleanliness and indifference may be the cause, but the fact that it often affects parts usually exempt, is opposed to such an opinion. Küchenmeister thinks that some endemic influence or constitutional peculiarity alone can explain the greatly exaggerated exudative process. It yields as readily to treatment as the ordinary forms.

*Diagnosis.*—We see, therefore, that scabies presents a varied combination of appearances capable of misleading any one who does not carefully analyze them. We must always bear in mind the importance of recognizing the primary phenomena of the disease, which furnish positive evidence in any particular case. We can not, therefore, make ourselves too familiar with all that pertains to the habits of the sarcoptes and its marks upon the skin, and even at the risk of repetition we shall explain the means to be employed in our examination. As has been remarked, the favorite spots for the burrows are the commissures and lateral surfaces of the fingers, the hands, penis, nates, feet, and folds of the axillæ, and in women the breasts, while in children we may find them equally distributed over the whole surface of the body. They may affect any part of it, in fact, except the head, although Hebra has cultivated them upon the face and scalp. It is the hand of the patient we first take up to examine, if we suspect the presence of scabies, for the collection of foreign matter, with which they continually come in contact, colors the burrows of a dark tint. If, therefore, we see streaks or spots which resemble such a burrow, we have only to rub it with some dark coloring matter, as ink, and then endeavor to wash it off, and if we really have to do with scabies we shall find that the pigment has penetrated beneath the epidermis and can not be removed. If we fail to find the burrows on the hands, for owing to some occupations these are at times an unpleasant dwelling place for the sarcoptes, we should examine the penis, where, from the yielding nature of the tissues and the undisturbed quiet, we find the most perfect specimens of animals, eggs, and burrows, or, in a person of sedentary habits, the buttocks. The specimen exhibited to the Society was snipped from the glans with a fine curved scissors by Hebra. This may be done without much pain to the patient, for it is not necessary to go deeply to cut out the whole burrow and animal intact. In persons who suffer from cold hands and feet we often find these parts entirely free, while the rest of the body may be covered with the eruption. This is corroborative of their love of heat, and when a person lies habitually on one side in bed, we sometimes find that side of the face affected, while that exposed to the cold remains untouched. Another exemplification of the same law may be seen in the immediate relief which a patient, wrought almost to frenzy by the itching of a general scabies at night, finds by jumping out of bed into the cold atmosphere of winter. The animal is best obtained from its burrow in the way above mentioned, or we may open the elevated track with a sharp knife or needle, and find it at its extremity. The males may be found with a lens upon the surface of the body, near the burrows of the females, or slightly embedded in the skin, appearing as a dark point. The young are generally found in fresh vesicles or in the epidermis undergoing their metamorphoses. We may often detect the lurking place by rubbing the suspected spots with German soap, and in twenty-four hours an exudation will follow and elevate the burrow. In this way we may often settle doubtful cases. By transporting a colony to one's arm the customs of the animal may be easily observed with a lens, and we see that they seldom or never go below the epidermis. In order to enter this, the acarus supports himself on his anterior end by means of its long posterior bristles, and works away with its lobster-like claws. It usually takes one-fourth or one-half an hour to penetrate the outer integument, but this once pierced its progress is more rapid. When coming in contact with the nerve papillæ it causes a stinging sensation. The poorer in nutriment the epidermis, the deeper it penetrates, and the greater the exudation caused, which lifts up the animal and gives the white color to the hole. The young brood seems to require the tender and last-formed epidermal layers for its food, and therefore bores deeper and produces more itching and exudation. When wandering about on the surface they produce no exudation whatever, but on the genitals and upon infants, where the epidermis is very thin, they are obliged to go deeper, whence the large exudation which ensues in such cases. It is a commonly received opinion that the parasite is to be found either within a vesicle, or, as it is said, a little way from it, without any apparent reference to the existence and importance of the burrow. In the majority of cases the vesicles have no connection with the position of the animal, and in the exceptional cases contain only the young, or are situated at one extreme end of this canal, while the game hunted is at the other.

Wilson has added to this confusion by giving, as one of the important diagnostic signs of scabies, "a peculiar scaliness and undermined state of the epidermis," from beneath which the animal may be extracted. This scaliness we have never observed, but we should literally as soon think of looking for a needle in a hay-mow as to attempt to extract an acarus until we had found a burrow, and a fresh one. In case none such can be found, we may still hope to obtain the proof we seek in the dried eggshells and fæcal masses the old burrows contain; or if this opportunity even is wanting, we may perhaps be fortunate enough to chance upon a vesicle which contains the young or a portion of its cast skin. Neither can we understand why it is that this writer should make the extraordinary statement that "one of the most remarkable phenomena of scabies is the localization of the acarus to the hands, while the eruption excited by it may be spread more or less extensively over the entire body." This is quite as incorrect as many of his statements with regard to vegetable parasitic affections. It is true that among the favorite lurking places of the animals are the folds of the skin between the fingers, for there they find the conditions of warmth and moisture they prefer; but, as we have seen, they affect as well the penis, folds of the axillæ and nates, and were we in search of a specimen we should turn first to these latter regions, where the burrows are less interfered with. Cases occur, however, and not unfrequently, where from the peculiar situation of the disease, or from interference either by the patient or by treatment, all immediate signs of the presence of the animals are wanting, where no burrow, fresh or dried, and not even a vesicle is to be found. Are we, in fact, able to recognize scabies by any peculiarities in the gross or secondary appearances? In the great majority of cases we are. We should examine carefully the whole body, notice the locality and character of the eruption, the appearances about the hands and feet, upon the penis and upon the nates, and if we have once thoroughly studied and understood the little peculiarities which give individuality to the multiple appearances of the disease, we shall readily recognize its nature, even when complicated by the simultaneous occurrence of an eczema or other cutaneous affection. We strongly insist upon the necessity of a careful attention to all these points, so important in diagnosis, on account of the great number of cases in which no other signs are present, and the frequency with which mistakes occur in consequence. We have repeatedly seen cases of well-marked scabies treated for a long time as some other disease, in which typical burrows were staring the physician in the face all the while, and again others which were equally unmistakable on close examination, though not so striking, called lichen or prurigo, when the microscope revealed the presence of the parasite. There is, in fact, an indescribable expression in the features of a case of scabies which can hardly be mistaken by any one who has once had the opportunity of seeing a large number of patients known to be affected with this disease. In cases of very long standing, or when confined to the hands, all specific appearances may be merged in those of chronic eczema, so that we may only suspect, not feel entirely sure in our diagnosis. In such cases we should always adapt our treatment to our suspicions, and at least remove all doubts with regard to the future character of the eruption by the application at once of our parasiticide.

*Ætiology.*—Scabies is found all over the world, but who had the first sarcoptes is a question not easily answered. It may have originated by transference from some other animal, for the same species is found parasitic on camels, lions and other animals in menageries, although it may have been man who affected the brute in such instances. We know that the mange of domestic animals, as the horse, pig, dog and cat, is capable of producing upon the skin of their keepers appearances similar to those of true scabies, but these are never of long duration, and yield much more readily to treatment than those produced by the sarcoptes hominis. We never see here in these days such cases of scabies as are met with in a European hospital. In Germany the old system of apprenticeship and its attendant wanderings through the land is still kept up, and in this way the disease is borne from one part of the country to another. Master workmen have generally but one bed for four apprentices, and the linen is seldom if ever changed. Scabies until recently has been comparatively rare here, although it has sometimes run through a school or an orphan asylum, or found its way into a good family, the young lady members of which often bear it about for a long time before suspecting its true nature. Of late years it has been quite largely introduced into the country by the crowds of immigrants which pour into our large cities. Every ship that arrives with such a freight brings scores of fresh cases of the disease, which spreads rapidly during the voyage, owing to the close quarters and want of cleanliness, and which serve as so many new centres of contagion wherever their inmates settle. The child carries it to the school and gives it to its playmates, and the older girl communicates it to the children or fellow servants at the place she takes. We have recently known an instance where it was started in a small manufacturing town but a few miles from Boston, and was introduced into a large number of families before its real nature was understood. Returned soldiers bring it frequently with them from the camp, and leave it behind as a constant reminder of their furlough. The mode of infection consists in the transfer of individuals from one host to another. This is accomplished in the majority of cases, without doubt, by the young and males, for, as we have seen, the mature female never leaves her hole. The acarus is an animal which loves the warmth, and on this account has been called nocturnal, but without good reason. The wandering and activity are excited only by the warmth imparted to the body by lying in bed, by sleeping with others, and by dancing, when hands are freely interchanged, and thus it is that the parasites are conveyed from one person to another. The nurse gives it to the child by placing her hand beneath its little nates, and so bearing it about. It may also be conveyed by wearing the clothes of any body afflicted with it, for both eggs and insects possess an obstinate vitality, and may exist months without food. It may happen occasionally that the old animal is scratched from its hole and thus transferred to another part of the same host or to a bed-fellow. We see, therefore, why it is that in some cases a person exposed to the disease may not exhibit any other symptoms than a solitary unnoticed burrow for weeks, until the young begin to make their appearance upon the surface, whereas in other cases the transference of the affection may be accomplished by several young or males, which begin their provoking rambles at once and cause an immediate eruption. It is this circumstance, principally, which determines the so-called period of incubation, and not, as Mr. Wilson says, the particular temperament, climate, season, age, confined air, etc. Scabies is seldom, if ever, caught by handling patients, however freely this may be done, and physicians and nurses are seldom infected. In one instance only, and that on an intensely hot day, did I ever see infection follow thousands of such contacts between patient and student. There must be many cases, however, where such transfer takes place without any result, for should several males or an unimpregnated female alone be conveyed to a fresh skin no general scabies would follow.

*Treatment.*—The chief indication in the treatment of scabies is of course to destroy the insect and its eggs, if possible. Any means which will accomplish this without injury to the skin and in the shortest time, is of course the best. The use of internal remedies belongs to the days when a belief in a dyscrasy prevailed, and nothing need be said about it. It will be needless to mention all the numerous remedies which have been used at various times and among different nations. Nearly everything has had a trial, but the remedy to kill the eggs as well as the insects is yet to be discovered. Among them all, one substance has attained a reputation which has made its use universal. Sulphur in some form, as a gas, as an acid, as a salt, in form of bath, or ointment, or tincture, either alone or mixed with other substances, is employed all the world over. There is, however, a great difference in the effect of the various preparations both upon the patient and upon the disease; and a knowledge of these effects is essential to a proper selection. Sulphur baths once employed, required weeks to produce any permanent effect, and were extremely disagreeable. Still less can be said in favor of the sulphur vapor bath, which, when used of sufficient strength to produce the desired results upon the parasite, usually causes so extensive an eczema as to require weeks of after-treatment for its removal. Simple sulphur mixed with fat (sulphur ointment) rubbed thoroughly into the skin, will in time cure the disease, but the process is a long one, and, when pushed to the extent to which the English use it, seldom fails of causing an artificial eczema. A more rational plan is to add to the preparation some substance which will more speedily remove the outer layers of epidermis, and allow the sulphur to come in direct contact with the animal. Such a substance is an alkali, and this combination in some form is at the base of all the various remedies and cures of the day. Among these, perhaps, Helmerich's ointment is the best, and it is this preparation which is employed by Hardy in his quick cure at San Louis Hospital. The original formula is:—Sulph. flor., ʒ ij.; pot. subcarb., ʒ i.; adipis, ʒ viij. Hardy uses lard, 300 parts; sulphur, 50 parts; and subcarbonate of potash, 25 parts; and his whole treatment lasts but an hour and a half. For the first half hour the patient is rubbed from head to heel with soft soap; during the second he is kept in a warm bath, and the third period is devoted to a universal friction with the above ointment. Bazin uses Helmerich's ointment unchanged, but employs two frictions instead of one. The patients are immediately dismissed, and are not again seen. In the majority of cases the quick cure is sufficient to overcome the specific character of the affection, but it by no means cures the other appearances which form so important a part of scabies, and relapses will frequently occur, for it is not easy to remove every egg at once. Three or four days is a much safer period to keep the patient under observation and treatment.

Another improvement suggested itself to Wilkinson, in the addition of some substance to prevent and heal the eczema by the use of sulphur in the above preparation, and some gritty matter to render the friction more effectual, and he accordingly added tar and chalk in the following formula:—sulph. venal., ol. fagi, sapon. domest., adipis, aa libram; cretæ, ℥iv.; ammon. hydrosulph., ℥ij. This ointment was modified by Hebra by the omission of the last ingredient as useless, and by a diminution in the quantity of sulphur to lessen the tendency to eczema, and for a long time he treated most successfully the great number of patients under his charge with the following preparation—sulph. venal., ol. fagi, aa ℥vi.; sapon. viridis, adipis, aa libram; cretæ, ℥iv. M. In cases where fat could not be employed, a more cleanly tincture was made by substituting alcohol to the same amount for the lard in the above formula. The action of this combination of remedies may be thus explained. The alkaline soap, assisted by the mechanical action of the chalk, removes the external layers of epidermis and opens the burrows, the sulphur acts as a parasiticide upon the animal, and the tar prevents the eczema, while the alcohol carries into the parts most deeply affected these active principles. The method pursued by Prof. Hebra until quite recently was as follows: The patient is put into a warm bath, where he remains for half an hour. Then a piece of coarse blanket is smeared with sapo viridis, and with it every finger and portion of the surface must be thoroughly rubbed. This is to be washed off in the bath and the skin to be dried. Then the ointment or tincture is thoroughly applied to the whole body, or in slight cases to the parts affected only, and the patient is carried to a warm chamber, where he remains until the next day, when the same process is repeated, and this treatment is pursued until itching no longer exists. Three baths are all that are allowed, unless many pustules are present, when more will be beneficial. The patient can be treated in this way at evening and continue at his work by day. In mild cases and tender skins, the modern sulphur soaps will be found of benefit, or we may use for ladies the following preparation introduced by Bourgignon: Ol. lavend., ol. menthæ, ol. carophyl., ol. cinnam., aa ℈i.; gummi tragacanth, ʒi.; potas. carb., ℥i.; lac. sulph., ℥iij.; glycerine, ℥vi. M. Within a few years, however, Professor Hebra has employed the Vleminckx solution entirely in the treatment of this disease. This method was introduced by the Surgeon-General of the Belgian Army, and its effect was found to be so satisfactory as to lead to its trial with the most eminent success in other cutaneous diseases. The formula for its preparation is as follows: Sulph. flor., ℥ij.; calcis vivæ, ℥i.; aquæ, ℥xx. Boil till ℥xxi. remain, and filter. We obtain in this way a clear orange-colored liquid, the expense of which is but very little. We get, accordingly, the necessary alkaline action, combined with the sulphur in a penetrating form, and the result is all that could be desired. The only objections to its use are its irritating effect and unpleasant odor, but the necessity for its use is limited to so short a period that they amount to little. It is applied in the following manner: The patient must rub his whole body thoroughly at night with a flannel cloth wet with the solution for half an hour. He then takes a warm bath of an hour's duration, during which the sulphur is washed from the skin, and subsequently a cool sponge bath of pure water. An application of some fatty substance or of a thin solution of oil of cade may be used if necessary.

One or two repetitions of this process will be sufficient to cure the most severe cases of scabies. We have employed this method of treatment repeatedly, and have never known the itching to fail to disappear almost entirely after the first application; but should it return at any spot, subsequent treatment may be confined to this locality.

Whichever of these three most deserving methods—that of the Helmerich's salve, of the Wilkinsonian ointment or tincture, or of the Vleminckx solution—we choose to employ, we must remember that there remains to be treated the secondary eruption, after the parasitic element has been eradicated. In mild cases it will disappear of itself, but in the severer and long standing forms it is necessary to apply the same principles of treatment which were mentioned in connection with eczema. We must also bear in mind the fact that a continuance of the specific treatment, after it has fulfilled its object, may of itself provoke appearances which the physician only creates while endeavoring to cure. The after treatment of scabies consists of an avoidance of all past sources of contagion and of baking in an oven the clothes previously worn.

### CHANGES IN THE KIDNEYS FROM LEAD POISON.

M. Lanceraux maintains that in the elimination of this poison a change altogether peculiar is produced in the kidneys—a true Bright's disease. The author alludes to cases of fatty degeneration of the kidneys in poisoning by phosphorus, by the acid nitrate of mercury, and by sulphuric acid. In the latter case he observed that "the cortical substance of the kidneys, of about normal consistence, was dotted with red. In the field of the microscope, there was absence of fat in the tubuli, but destruction of most of the epithelial cells, which formed a finely-granular grayish mass. The walls of the canaliculi appeared intact, but the interstitial connective-substance was altered, and in course of proliferation (nephritis)." The author says that the renal affection co-existent with lead poisoning is not a simple coincidence—that he has always found its characters very analogous, if not identical. He thus sums up the results of his investigations in three cases:

There was, 1st, an advanced stage of lead poisoning, with cachexy; 2d, a lesion of the kidneys, always characterized by inequality of their surface; atrophy of their cortical substance; hyperplasy of the connective substance; destruction, or even the disappearance of the cellular elements, with albumen present in the urine. This renal affection only succeeds to an advanced stage of lead disease.—*Am. Journal of the Med. Sciences*, from *Brit. and For. Med.-Chir. Rev.*, April, 1864, from *L'Union Médicale*, Dec. 15, 1863.

